# Integrated Analysis of DNA Methylation and Gene Expression Profiles in a Rat Model of Osteoarthritis

**DOI:** 10.3390/ijms25010594

**Published:** 2024-01-02

**Authors:** Jin Mi Chun, Joong-Sun Kim, Chul Kim

**Affiliations:** 1 Digital Health Research Division, Korea Institute of Oriental Medicine, Yuseong-daero 1672, Daejeon 34054, Republic of Korea; jmchun@kiom.re.kr; 2 College of Veterinary Medicine, Chonnam National University, Gwangju 61186, Republic of Korea; 3 KM Data Division, Korea Institute of Oriental Medicine, Daejeon 34054, Republic of Korea

**Keywords:** osteoarthritis, gene expression, DNA methylation, ITGA2, Erk1/2

## Abstract

Osteoarthritis (OA) is common and affected by several factors, such as age, weight, sex, and genetics. The pathogenesis of OA remains unclear. Therefore, using a rat model of monosodium iodoacetate (MIA)-induced OA, we examined genomic-wide DNA methylation using methyl-seq and characterized the transcriptome using RNA-seq in the articular cartilage tissue from a negative control (NC) and MIA-induced rats. We identified 170 genes (100 hypomethylated and upregulated genes and 70 hypermethylated and downregulated genes) regulated by DNA methylation in OA. DNA methylation-regulated genes were enriched in functions related to focal adhesion, extracellular matrix (ECM)-receptor interaction and the PI3K-Akt and Hippo signaling pathways. Functions related to extracellular matrix organization, extracellular matrix proteoglycans, and collagen formation were involved in OA. A molecular and protein-protein network was constructed using methylated expression-correlated genes. Erk1/2 was a downstream target of OA-induced changes in DNA methylation and RNA expression. We found that the integrin subunit alpha 2 (ITGA2) gene is important in focal adhesion, alpha6-beta4 integrin signaling, and the inflammatory response pathway in OA. Overall, gene expression changes because DNA methylation influences OA pathogenesis. ITGA2, whose gene expression changes are regulated by DNA methylation during OA onset, is a candidate gene. Our findings provide insights into the epigenetic targets of OA processes in rats.

## 1. Introduction

Osteoarthritis (OA) is the most common degenerative joint disease in the elderly; it affects more than 250 million people worldwide, causing pain and physical disability, leading to decreased joint movement [1,2,3]. Due to population aging, the impact may increase exponentially over time [4]. The incidence of OA is influenced by many factors, such as work, sports, musculoskeletal injuries, obesity, sex, genetics, joint disorders, environmental and lifestyle factors, metabolic disorders and diseases, and chronic inflammation [5]. Considering the various risk factors of OA, recent research is aimed at diagnosing and monitoring the progression of OA through multifaceted efforts, such as examining the effects of arthroscopic microfracture, radiographic biomarkers, and vibroacoustic diagnostics [6,7,8]. Based on recent findings, OA involves metabolic processes and epigenetic modifications, in addition to cellular and molecular mechanisms of inflammation in its progression and pathogenesis [4,5]. The treatment of OA is difficult, and the etiology and pathological mechanisms are not yet fully understood; however, new attempts are currently being made to understand the pathogenesis of OA [9]. In addition, recent studies have reported the regulation of major biomarkers, pathological signaling pathways such as Wnt/β-catenin, NF-κb, focal adhesion, HIFs, TGFβ/ΒΜP and FGF, and the key regulators AMPK, mTOR, and RUNX2, in the development and progression of OA [10,11,12].

Owing to recent advances in next-generation sequencing (NGS) technology, several studies have investigated the molecular mechanisms underlying various diseases, including cancer, infectious diseases, and inherited genetic diseases [13]. For example, integrative analysis of gene expression and DNA methylation profiles aids in identifying DNA methylation-induced changes in gene expression in different disease states, such as cancer, acute myocardial infarction, and Kashin-Beck disease [14,15,16]. This technology offers new ways to understand the significance of DNA methylation and is used to identify potential biomarkers for the early diagnosis of several diseases. Recently, the mechanisms underlying the pathogenesis of OA have been studied to identify target genes responsible for OA development using single-omics analyses [17,18,19,20].

Because multiple factors mediate OA, integrated system approaches are needed to understand the overall molecular mechanisms and etiology and to better guide the development of new treatment strategies for OA [21]. Omic techniques for target discovery include transcriptomics, proteomics, and epigenomics. These approaches map RNA, protein, and epigenomic modifications to different cell types and tissues in humans and model organisms. They can be used to compare health and disease statuses to better understand the mechanisms underlying OA, guide the development of new therapeutic strategies, and enable us to understand the molecular mechanisms of OA pathogenesis [21,22]. Among them, DNA methylation is one of the most studied epigenetic modifications that plays an important role in regulating gene expression. Because DNA methylation analysis can provide valuable insights into gene regulation and identify potential biomarkers, genome-wide DNA methylation using methyl-seq would be a more suitable method to obtain detailed information in coding and intergenic regions [23]. In particular, DNA methylation of gene promoter regions may be associated with the repression of gene transcription during OA pathogenesis and may serve as a therapeutic target [24].

Although aberrant changes in DNA methylation have been implicated in the pathogenesis of many diseases, such as cancer and metabolic syndrome [25,26], the molecular mechanisms underlying the interactions between DNA methylation and gene expression, particularly in the pathogenesis of OA, are under researched. Therefore, to identify gene expression changes regulated by DNA methylation during OA pathogenesis, we performed methyl-seq and RNA-seq in a monosodium iodoacetate (MIA)-induced OA rat model to profile genome-wide DNA methylation and gene expression, respectively. We also constructed an integrated network of DNA methylation and expression, which revealed epigenetically regulated genes associated with functional enrichment, and performed network analysis of putative candidate genes.

## 2. Results

### 2.1. Transcriptomic Changes in the MIA-Induced Rat Model of OA

Samples were collected from rats housed in negative control (NC) and MIA-injected groups. We verified whether MIA, which is a sign of OA, was induced, as described in our previous study. Our previous results showed that the delta knee joint diameter, an index of joint edema, in MIA-induced rats continued to increase throughout the experimental period compared to the NC group, confirming that joint swelling was significantly increased by MIA [27,28].

**Figure 1:**
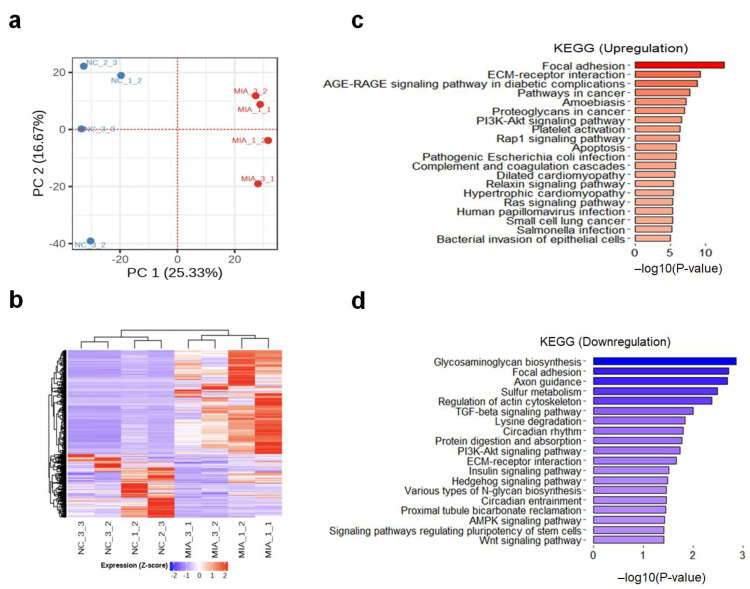
Gene expression profiles of a monosodium iodoacetate (MIA)-induced osteoarthritis (OA) rat model. (**a**) Principal component analysis (PCA) plot of gene expression profiling in NC vs. MIA samples. (**b**) Heatmap of differential expressed genes between NC and MIA. (**c**,**d**) Top 20 functional annotation results using the Kyoto Encyclopedia of Genes and Genomes pathway of up/downregulated genes. NC, negative control; MIA, only MIA-induced.

To identify changes in gene expression in rats with MIA-induced OA, RNA-seq analysis was performed. Using principal component analysis (PCA) and hierarchical clustering, the NC- and MIA-treated rats (n = four per group) were separated into two groups (Figure 1a). Based on our criteria of a log2 (fold change, FC) ≥ |1| and false discovery rate (FDR) < 0.05, we identified 1677 differentially expressed genes (DEGs) between the NC and MIA-induced OA samples (1044 upregulated and 633 downregulated; Figure 1b, Table S1). As shown in the heatmap of DEGs between the NC and MIA groups, the number of up-regulated genes was greater than that of the down-regulated genes, and the expression of many genes was affected by MIA.

To examine the functional annotation of genes, we performed a gene set enrichment analysis (GSEA) of both the up- and down-regulated genes following MIA treatment. Genes were enriched in functions involved in ‘focal adhesion’, ‘ECM-receptor interaction’, and the ‘PI3K-Akt signaling pathway’ according to the Kyoto Encyclopedia of Genes and Genomes (KEGG) pathway analysis; these terms all overlapped between the up- and down-regulated genes, indicating that genes with different patterns play roles in regulating the same functions (Figure 1c,d). Downregulated genes were enriched in signaling pathways, such as TGF-beta (*p*-value = 0.009), Hedgehog (*p*-value = 0.03), and Wnt (*p*-value = 0.03). In the REACTOME pathways, as shown in Table S1, upregulated genes were enriched in extracellular matrix organization (*p*-value = 3.18 × 10^−13^) and ECM proteoglycans (*p*-value = 3.71 × 10^−6^), and downregulated genes were enriched in the extracellular matrix, such as collagen biosynthesis and modifying enzymes (*p*-value = 4.58 × 10^−5^), collagen formation (*p*-value = 5.51 × 10^−4^), extracellular matrix organization (*p*-value = 0.001), collagen chain trimerization (*p*-value = 0.002), and glycosaminoglycan metabolism (*p*-value = 0.0048). Therefore, DEGs regulated by MIA affect the mechanisms of the disease through multiple enrichment pathways.

### 2.2. Genome-Wide DNA Methylation Profiling of OA

**Figure 2:**
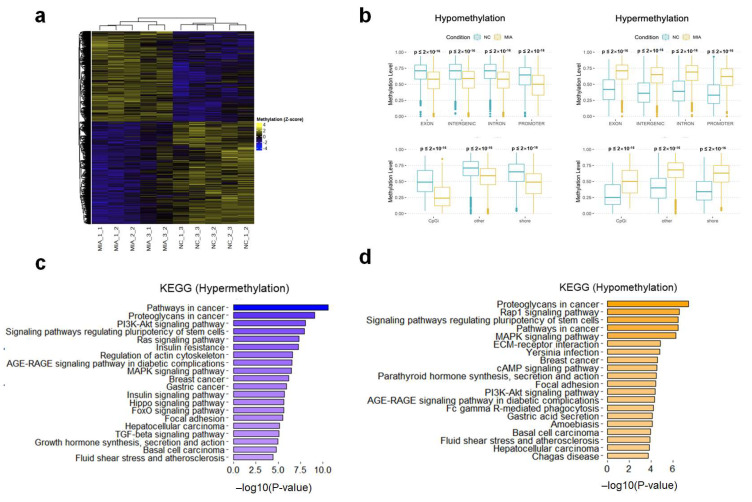
Changes in DNA methylation in a monosodium iodoacetate (MIA)-induced osteoarthritis (OA) rat model. (**a**) Heatmap of differential methylated regions between NC and MIA groups. (**b**) Boxplot of the methylation levels of genomic regions and CpG categories. (**c**,**d**) Pathway enrichment using the Kyoto Encyclopedia of Genes and Genomes pathway gene sets of hyper and hypomethylated genes. NC, negative control; MIA, only MIA-induced.

We identified 39,749 differentially methylated regions (DMRs, window size = 1000 bp), corresponding to 4101 (hypermethylated) and 3806 (hypomethylated) genes (Figure 2a, Table S2). We annotated the genomic regions and CpG information in the DMRs for each methylation status. Our methylation profile showed a clear difference in the levels of methylation in various genomic regions between the NC and MIA-induced OA rat models (Figure 2b). We next performed KEGG/REACTOME pathway enrichment on differentially methylated genes (DMGs). The functional annotation results of the KEGG pathways are shown in Figure 2c,d, and the results of the REACTOME pathway are shown in Table S2. Hypermethylated genes were enriched in signaling by the receptor tyrosine kinases (*p*-value = 7.85 × 10^−9^), focal adhesion (*p*-value = 3.27 × 10^−6^), and extracellular matrix organization (*p*-value = 8.58 × 10^−5^), as well as the PI3K-Akt (*p*-value = 9.15 × 10^−9^) and the MAPK signaling pathways (*p*-value = 3.18 × 10^−7^, Figure 2c and Table S2). Hypomethylated genes were enriched in extracellular matrix organization (*p*-value = 4.04 × 10^−9^), ECM-receptor interactions (*p*-value = 1.43 × 10^−5^), and focal adhesion (*p*-value = 3.73 × 10^−5^), as well as the MAPK (*p*-value = 5.58 × 10^−7^) and PI3K-Akt signaling pathways (*p*-value = 4.15 × 10^−5^, Figure 2d and Table S2). We also observed overlapping in the functions of hyper/hypo-regulated genes, in the functional terms of ‘focal adhesion’, the ‘MAPK signaling pathway’, the ‘PI3K-Akt signaling pathway’, ‘signal transduction’, ‘signaling by receptor tyrosine kinases’, and ‘extracellular matrix organization’. These results suggest that instead of a sporadic occurrence, DNA methylation occurs in specific pathways and may play a role in gene expression.

### 2.3. Dysregulated Gene Expression Induced by DNA Methylation

**Figure 3:**
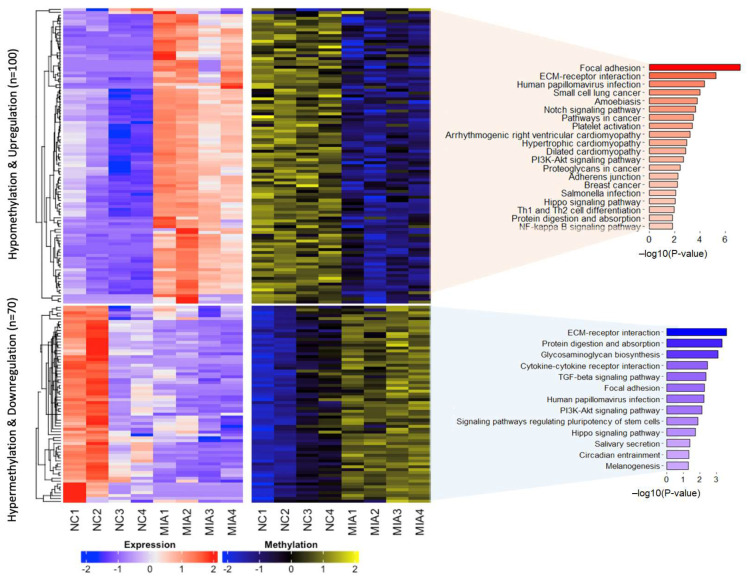
Heatmap of DNA methylation derived dysregulated gene expression with functional annotation. Inversely correlated genes (n = 170) between DNA methylation and gene expression are shown. Expression (low: blue and high: red) and methylation (hypo: blue and hyper: yellow) values were scaled according to the samples was scaled by samples.

To identify the genes regulated by DNA methylation in MIA-induced OA, we examined RNA-seq data for negative correlations between DNA methylation and gene expression. We focused on the differences in methylation patterns between NC and MIA-induced rats. We identified 170 genes, corresponding to 100 hypomethylated and upregulated genes (Table S3) and 70 hypermethylated and downregulated genes (Table S4), with a significant inverse correlation between gene expression and DNA methylation (Figure 3). As shown in the functional annotation results of the KEGG pathways (Figure 3) and the REACTOME pathways (Table S3), hypomethylated and upregulated genes were significantly enriched in focal adhesion (*p*-value = 6.63 × 10^−8^), ECM-receptor interactions (*p*-value = 5.22 × 10^−6^), PI3K-Akt signaling pathway (*p*-value = 0.002), extracellular matrix organization (*p*-value = 2.41 × 10^−8^), collagen formation (*p*-value = 8.86 × 10^−5^), and ECM proteoglycans (*p*-value = 1.64 × 10^−4^, Table S3). In contrast, hypermethylated and downregulated genes were enriched in functions related to ECM-receptor interactions (*p*-value = 2.57 × 10^−4^), glycosaminoglycan biosynthesis (*p*-value = 8.48 × 10^−4^), cytokine-cytokine receptor interactions (*p*-value = 0.003), focal adhesion (*p*-value = 0.005), and the PI3K-Akt signaling pathway (*p*-value = 0.008). These results showed overlap in the functions of DNA methylation-regulated genes, in the functional terms of ‘focal adhesion’, ‘ECM-receptor interaction’, ‘PI3K-Akt signaling pathway’, and ‘Hippo signaling pathway’ by KEGG pathways. In the REACTOME pathway analysis (Table S4), candidate genes were involved in functions related to ‘extracellular matrix organization’, ‘ECM proteoglycans’, and ‘collagen formation’ in OA, indicating that genes with different patterns play a major role in regulating the same function. These results suggest that DNA methylation-regulated genes may play a role in specific functions and pathways in OA.

### 2.4. Functional Network Analysis of OA Candidate Gene

**Figure 4:**
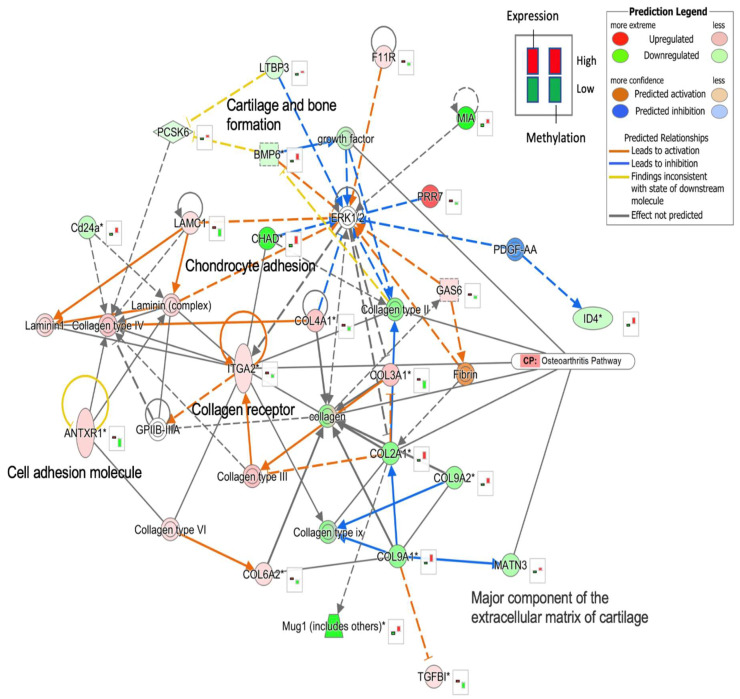
Inference of the gene network from methylation-expression correlated genes using Ingenuity Pathway Analysis. The network with the highest IPA score was constructed using the IPA Network Module. Molecular relationships were predicted using the MAP function of the IPA overlay function. The right-hand boxes of the genes represent expression (left bar) and methylation (right bar) values. Dotted line indicates indirect activation or inhibition. IPA, Ingenuity Pathway Analysis.

For the interactions between OA candidate genes induced by MIA, we performed an integrated network analysis of DNA methylation and gene expression following MIA induction. We used Ingenuity Pathway Analysis (IPA, QIAGEN Inc., Venlo, The Netherlands) to construct a network using the 170 candidate genes. Functional network analysis of genes regulated by DNA methylation showed that cartilage and bone formation, a major component of the extracellular matrix of cartilage, collagen receptors, chondrocyte adhesion, and cell adhesion molecule, are related to each other and are involved in the OA pathway. Among the candidate genes, we found that the growth factor, collagen type II, integrin alpha 2 (*ITGA2*, a collagen receptor), collagen, and *MATN3* (a major component of extracellular matrix of cartilage) were associated with the canonical OA pathway (Figure 4). Lamin-related genes and collagen types III, IV, and VI (*COL3A1*, *COL4A1*, and *COL6A2*, respectively) were upregulated and hypomethylated, while collagen types II and IX (*COL2A1*, *COL9A2*, and *COL9A1*, respectively) were downregulated/hypermethylated [29]. The ERK1/2 complex was downstream of the genes involved in methylation and expression in OA. However, we could not predict the molecular activity of ERK1/2 because of overlapping activation and inhibition signals.

**Figure 5:**
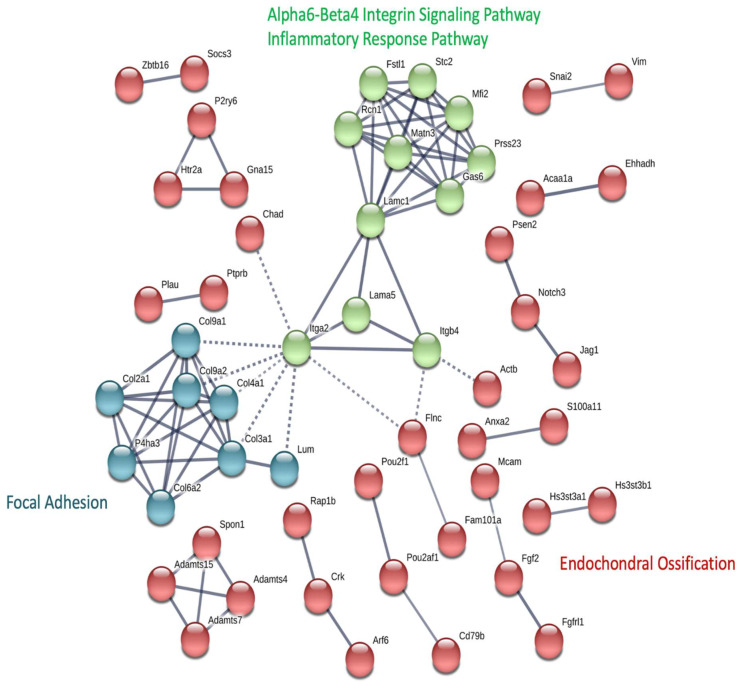
Protein-protein interaction (PPI) network of methylation-expression candidate genes. The PPI network of 170 candidate genes were clustered using the k-means clustering method (k = 3). Disconnected single nodes are removed. Dashed lines represent inter-cluster (connected with other clusters) edges.

We predicted the protein-protein interaction (PPI) of candidate genes (Figure 5). The PPI network was divided into three clusters using K-means clustering. Cluster 1 (red) consists of several sub-networks and is related to endochondral ossification. Cluster 2 (green) is related to alpha6-beta4 integrin signaling and the inflammatory response pathways. Cluster 3 comprised collagen genes involved in focal adhesion. ITGA2 (integrin subunit alpha 2, inter-cluster edge) may be an important core mediator among the clusters.

## 3. Discussion

Current research suggests that changes in gene expression or methylation are involved in the pathogenesis of OA; however, multi-omics analysis remains under researched [17,30,31]. Functional genomic approaches to OA focus on genome-wide DNA methylation and gene expression profiling, allowing for the identification of new biomarkers involved in the pathogenesis of OA [32]. Therefore, we sought to identify the molecular mechanisms underlying the interaction between DNA methylation and gene expression in OA. We profiled genome-wide DNA methylation and gene expression in an MIA-induced OA rat model and performed an integrated network of DNA methylation to reveal epigenetically regulated genes whose expression was associated with functional enrichment and network analysis of putative candidate genes.

We investigated changes in gene expression due to DNA methylation in OA and the underlying biological mechanisms. A multi-omics analysis was performed using an MIA-induced OA rat model. Our MIA-induced model is a chemically induced OA animal model that has characteristics similar to human OA and has rapid onset and minimal invasiveness [33,34]. Gene expression and methylation differed significantly between NC and MIA-induced OA rat model groups. We detected more upregulated and hypomethylated genes than previously reported [20,31,35]. The PCA results of the gene expression profiles showed that the NC and MIA groups were classified by PC1 (25.33%). We identified enriched DEGs that were involved in ‘focal adhesion’, ‘ECM-receptor interaction’, the PI3K-Akt signaling pathway, and the extracellular matrix related to functions of cartilage actively remodeled by chondrocytes in OA. Therefore, DEGs regulated by MIA function affect the mechanisms of the disease through multiple enrichment pathways. The pathway enrichment analysis of the DMGs revealed that the functional terms ‘focal adhesion’, ‘MAPK signaling pathway’, ‘PI3K-Akt signaling pathway’, ‘signal transduction’, ‘signaling by receptor tyrosine kinases’, and ‘extracellular matrix organization’ were significantly enriched. Therefore, the signaling pathways (e.g., MAPK, PI3K-Akt, cAMP, FoxO, and Ras) are activated and/or inhibited in OA. Signaling pathways may play an important role in the pathogenesis of OA (e.g., apoptosis, TGF-β, NF-κB, and Wnt) [36,37,38,39]. Our methylation results suggest that epigenetic regulation of signaling pathways is involved in the development of OA. Recent studies have shown that DNA methylation can affect the expression of genes involved in OA pathogenesis [24].

We focused on 170 candidate genes whose expression was regulated by DNA methylation in OA. DNA methylation-regulated genes are strongly associated with focal adhesion, ECM-receptor interaction, PI3K-Akt, and the Hippo signaling pathway. Functions related to ‘extracellular matrix organization’, ‘ECM proteoglycans’, and ‘collagen formation’ are involved in OA, as shown in our expression and/or methylation profiles. These results suggest that DNA methylation-regulated genes may play a role in specific functions and pathways involved in the pathogenesis of OA. In particular, PI3K-Akt signaling mediates synovial inflammation, subchondral osteosclerosis, ECM homeostasis, chondrocyte proliferation, apoptosis, autophagy, and inflammation greatly influencing cell fate and OA pathophysiology. It has the potential as a PI3K/AKT/mTOR signal-related inhibitor and modulator of OA [40].

Network analysis revealed that Erk1/2 was a putative target molecule downstream of the candidate genes. The molecular activity of Erk1/2 was not predicted in our network analysis, but Erk1/2 activation may be involved in IL-1β-mediated MMP3, MMP13, type II collagen, and aggrecan expression in human chondrocytes [41]. Additionally, Erk, which is activated by inflammatory cytokines, is involved in OA pathogenesis by mediating cellular responses to intracellular signaling molecules [42]. MEK/ERK inhibitors have been shown to inhibit metalloproteinases and inflammatory activities, and the ERK/MAPK pathway has been proposed as a therapeutic target for OA [43]. This implies that DNA methylation affects numerous genes that are functionally related to cell adhesion, the extracellular matrix of cartilage, and cartilage formation, and ultimately affects the Erk1/2 signaling pathway. In a genome-wide DNA methylation study, differential methylation of genes involved in ERK/MAPK, PI3K, and NFAT signaling that undergo damage-associated methylation changes were identified in subchondral bone and cartilage [44]. We also identified the most closely connected hub genes in an integrated network of DNA methylation and gene expression. ITGA2 showed an upregulated/hypomethylated expression pattern in NC vs. MIA-induced rats, which plays a role in the collagen adhesion of platelets and other cells, regulation of collagen and collagenase gene expression, and force generation and organization of the newly synthesized extracellular matrix [45]. These results indicated that methylation changes in hub genes play an important role in the regulation of OA-induced processes. Furthermore, ITGA2 was predicted to be a core gene from PPI network analysis. The ITGA2 gene is a membrane glycoprotein and represents one of the collagen receptors on the cell surface [46]. It is involved in focal adhesion, integrin signaling, the inflammatory response pathway, and endochondral ossification (Figure 5) and appears to play the most important role in OA pathogenesis. miR-140 downregulates ITGA2 levels and inflammatory cartilage degradation in rheumatoid, which supports our findings [41,47]. However, conflicting results have been also reported [48]. Consistent with our results, a previous report suggested that ITGA2 is a potential candidate biomarker of OA [48]. This gene is involved in cell–cell adhesion and may play a role in inflammation, fibrosis, and collagen binding [49].

Recently, studies on the epigenetic mechanism using DNA methylation profiling of natural products have been reported [50,51]. The candidate genes and pathways derived from our results need to be applied to these studies to verify biomarkers of OA through the DNA methylation mechanism. Our study has several limitations. The sample size was small, predictions of the biomarkers and signaling, selected based on our results, require confirmation, and the suggestion of treating OA using these biomarkers must be supported by more research, which may determine whether these biomarkers are regulated. Multi-omics analyses and research on other epigenetic mechanisms related to OA pathogenesis are required.

Overall, we performed an integrative analysis of DNA methylation and gene expression in an MIA-induced OA rat model. Our results will help to elucidate the pathogenesis of OA; further, we have suggested new candidate target genes mediated by DNA methylation in OA. These potential biomarkers provide basic data on the epigenetic mechanisms of OA and promote its early diagnosis and treatment.

## 4. Materials and Methods

### 4.1. Monosodium Iodoacetate (MIA)-Induced Rat Model

Our experimental model was the MIA-induced OA rat model described previously [34], and the experimental procedure has been described in detail in a previous report [27,28]. In this study, 7-week-old male Sprague Dawley rats (Daehan Bio Link, Inc. Eumseong, Republic of Korea) were acclimatized to a 1:1 light/dark cycle with air conditioning for one week. Afterwards, the rats were randomly assigned to either the untreated saline group (negative control, NC) or the MIA injected with saline (MIA) group (n = five per group). For MIA induction, the right knee joint of the rats was shaved under isoflurane inhalation anesthesia and MIA (3 mg/50 μL saline) was directly injected into the intra-articular space of the right knee of MIA-treated rats on day 1. The MIA concentration was selected based on a previous study [52]. After MIA was induced, the knee diameter, which can be used to measure the inflammatory index of joint swelling, was measured once a week for 21 days to observe the symptoms of OA. On day 22, the animals were sacrificed, and the articular cartilage was carefully dissected away from the femur and tibia of the knee joint tissue and rapidly isolated for RNA-seq and DNA methylation sequencing analysis, immediately frozen in liquid nitrogen, and stored at −70 °C until analysis. All animal experimental procedures were approved by the Ethics Committee of Kyungpook National University (approval no. KNU 2018-0091).

### 4.2. RNA Extraction and Quality Control

Total RNA from each tissue was extracted using a mirVana™ miRNA Isolation Kit (Life Technologies, Grand Island, NY, USA) according to the manufacturer’s protocols. Total RNA from the purified samples was treated with DNase, and RNA purity was measured using a NanoDrop 8000 spectrophotometer (Thermo Fisher Scientific, Wilmington, DE, USA). For RNA-sequencing, RNA integrity was confirmed using an Agilent Technologies 2100 Bioanalyzer (Agilent Technologies, Santa Clara, CA, USA), and samples with an RNA Integrity Number (RIN) value > 7.0 were used.

### 4.3. RNA-Sequencing Analysis

RNA-seq libraries were prepared using the TruSeq Stranded Total RNA H/M/R Prep Kit (Illumina, San Diego, CA, USA) according to the manufacturer’s instructions and sequenced on the Novaseq 6000 (Illumina, San Diego, CA, USA) to generate 101 bp paired-end reads. Reads were trimmed to remove adapters and low-quality reads (Phred quality < 20) using the Trimmomatic [53]. High-quality reads were mapped to the *Rattus norvegicus* genome (Rnor 6.0) using HISAT2 (v.2.1.0) [54], and gene expression levels were quantified using the ballgown R package (v. 2.12.0) [55]. After the sample quality check, we removed one low-quality sample per group based on the PCA and read count distribution (n = four per group). Genes that were differentially expressed between the two groups were identified using DESeq2, which is based on negative binomial distribution models using raw count data [56]. DEGs were considered with a cut-off threshold of log2 FC ≥ |1| and FDR < 0.05.

### 4.4. DNA Extraction and Methyl-Sequencing Analysis

Genomic DNA was extracted from articular cartilage tissues using a QuickGene DNA Tissue Kit S (KURABO, Osaka, Japan) following the manufacturer’s instructions. DNA quantification was performed as a quality check, and four samples per group were selected for library construction and sequencing. DNA libraries were prepared using a SureSelect Rat Methyl-Seq Kit (Agilent, Santa Clara, CA, USA) according to the manufacturer’s instructions. Isolated genomic DNA was used for library preparation and was bisulfite conversion using an EZ DNA Methylation Gold Kit (Zymo Research, Carlsbad, CA, USA). The resulting methyl-seq library was sequenced on a Novaseq 6000 (Illumina, San Diego, CA, USA) in the 101bp paired-end mode. Read trimming was performed using Trimmomatic software (v0.38). The trimmed reads were mapped to the bisulfite-converted *Rattus norvegicus* genome using Bismark (v.0.20.0) [57]. The methylKit R package (v. 1.6.0) was used for analysis of methyl-seq data and DMR identification (window size: 1000 bp and step size: 500 bp) [58]. Regions with two or more CpGs, ≥ 10% mean methylation difference and Q-value < 0.01, were considered significant. The MethylKit software (v1.10.0) was used to annotate peaks and assess the distribution of methylation peaks across genomic features. Genomic features were classified into six types of regions (intergenic, 5′ UTR, 3′ UTR, promoter, CDS and intron) based on the UCSC genome annotation.

### 4.5. Functional Annotation and Enrichment Analysis

GSEA was used to assess the functional effects of DNA methylation on gene regulation using predetermined sets of genes. GSEA of differentially expressed and methylated genes was performed using enrichR [59]. KEGG and REACTOME gene sets were used for gene set analysis. Statistical significance was set at *p* < 0.05, and the top 20 genes were selected and plotted.

### 4.6. Selection of Candidate Genes Based on DNA Methylation and Expression Levels

To investigate candidate genes that showed an inverse correlation between DNA methylation and expression, we calculated the Pearson correlation coefficient and considered it significant at *p* < 0.05.

### 4.7. Network Inference Using Ingenuity Pathway Analysis (IPA) and STRING

The molecular network of the candidate genes was constructed using IPA (QIAGEN Inc., http://www.ingenuity.com). The data matrix of gene expression and methylation of the candidates was uploaded to IPA and then core analysis function was performed. Using the molecular activity prediction (MAP) function of IPA, we constructed a network using genes with a negative correlation between expression and methylation and predicted the relationship between genes. The network with the highest IPA score among the generated networks was selected and connected to a canonical OA pathway.

## 5. Conclusions

In this study, we performed an integrative analysis of DNA methylation and gene expression in an MIA-induced OA rat model. The analysis revealed 170 genes (100 hypomethylated and upregulated genes and 70 hypermethylated and downregulated genes) and integrated networks involved in methylation and gene expression. A molecular and protein-protein network was constructed using the methylated expression-correlated genes. Erk1/2 was a downstream target of OA-induced changes in DNA methylation and RNA expression. We found that the ITGA2 gene is important for focal adhesion, alpha6-beta4 integrin signaling, and the inflammatory response pathway in OA. Further, gene expression changes due to DNA methylation influenced the pathogenesis of OA. Overall, we identified several epigenetically regulated genes and pathways involved in the onset of OA. Our findings provide insights into epigenetic targets of OA processes in rats.

## Data Availability

The complete sequences generated in this study were deposited in the SRA repository under the accession number of PRJNA717328.

## Supplementary Materials

The following supporting information can be downloaded at: https://www.mdpi.com/article/10.3390/ijms25010594/s1.
